# Complicated Community-Acquired Staphylococcus Endocarditis and Multiple Lung Abscesses: Case Report and Review of Literature

**DOI:** 10.1155/2011/981316

**Published:** 2011-09-22

**Authors:** Musa A. Garbati, Imad M. Tleyjeh, Abdullah A. Abba

**Affiliations:** ^1^Division of Infectious Diseases, Department of Medicine, King Fahad Medical City, Riyadh 11525, Saudi Arabia; ^2^Division of Pulmonology, Department of Medicine, Ahmadu Bello University, Zaria, Nigeria

## Abstract

*Background*. Isolated tricuspid valve endocarditis in the absence of risk factors in the community setting is very rare and can be easily missed in patients with hitherto normal valves. *Case Presentation*. We present a case of a 49 year old gentleman who presented with generalized body aches, fever, and jaundice and was initial diagnosed as hepatitis. He subsequently developed recurrent episodes of panic attacks and shortness of breath and later multiple skin abscesses. Further investigations excluded pulmonary embolism but revealed multiple abscesses in the body including the lungs. Blood cultures and culture from abscesses grew *S. aureus.* An initial transthoracic echocardiogram was normal. A transesophageal echocardiogram subsequently confirmed endocarditis on a normal natural tricuspid valve and multiple lung abscesses. He was successfully treated with appropriate antibiotics. *Conclusion*. We discuss the pathogenesis of this patient's presentation highlight the need for assessment and proper evaluation of patients with unexplained bacteremia.

## 1. Introduction

*Staphylococcus aureus* is an important cause of community-acquired bacteremia and is associated with substantial morbidity and mortality [1–3]. A recent population-based surveillance study reported *Staphylococcus aureus *as being the most common cause of nosocomial bacteremia and the second most common cause of community-acquired bacteremia [4]. An earlier retrospective survey from the same population (Olmsted County, Minnesota) which is said to be a relatively isolated community over a period of 30 years (1970–2000) by Tleyjeh et al. [5] also revealed that *S. aureus* (26%) was second only to viridians group streptococci (44%) as the principal causative agent of infective endocarditis. However, Fowler and colleagues [6] undertook a prospective analysis of *S. aureus* endocarditis cases seen at 39 medical centers in 16 countries from June 2000 through December 2003 and reported that *Staphylococcus aureus* was the pathogen identified most frequently. 

*Staphylococcus aureus* owes its virulence to the presence of toxins (alpha-toxin, beta-toxin, gamma-toxin, delta-toxin, exfoliatin, enterotoxins, Panton-Valentine leukocidin (PVL), and toxic shock syndrome toxin-1 (TSST-1)) [7]. Up to 80% of people are colonized with *S. aureus*; most only intermittently but up to 20–30% are persistently colonized. Colonization rates in health care workers, persons with diabetes, and patients on dialysis are more than in the general population. The anterior nares are the predominant sites of colonization in adults; carriage here has been associated with the development of bacteremia [8]. Patients with SAB can develop a broad array of complications which may be difficult to recognize and often lead to disability or death. Complications of SAB are common; frequencies range from 11–53% [9, 10] and can occur in almost any body site. However, certain complications are especially important because they are difficult to diagnose and are often associated with poor outcome. Risk factors associated with development of complications due to SAB vary depending on the route of acquisition, site of infection, presence or absence of foreign material, pathogen characteristics, and host predisposition. Patients with community-acquired SAB have an increased risk for metastatic complications [7, 11–15].

For decades, infective endocarditis (IE) caused by *Staphylococcus aureus *has been viewed primarily as a community-acquired disease especially associated with injection drug use (IDU) [15–21]. However, this syndrome has continued to be a frequent clinical problem even in communities where IDU is relatively uncommon. Its epidemiologic characteristics seem to remain stable despite the advent/increase in the frequency of multidrug-resistant organisms in both the health-care and community environments as revealed by an earlier study [11]. A comparison of nosocomial and community-acquired SAB reveals several fundamental differences. Community-acquired SAB frequently develops in the absence of a primary focus of infection and is more likely to result in endocarditis and secondary metastatic foci of clinical infection. In contrast, nosocomial SAB tends to be diagnosed earlier, a primary site of portal of entry is usually identified and endocarditis is less frequent as are secondary foci of infection. Irrespective of the epidemiological origin of SAB, the mortality remains high particularly when nosocomially acquired, where the presence of severe underlying disease and other comorbidities contribute to the high mortality.

The overall incidence of involvement of the tricuspid valve in patients with infective endocarditis is in the range of 5% to 10%, with up to 80% of tricuspid valve endocarditis occurring in drug addicts [11]. Right-sided endocarditis is a clinical rarity in nondrug addicts without previous heart disease, and its diagnosis sometimes presents a significant clinical challenge [22]. Right-sided IE occurs much less frequently in nonaddicted patients as a complication of permanent intravenous catheter placement, untreated skin or genital infections [23–25], or underlying congenital heart disease [26]. In these patients, endocarditis arising from *S. aureus *primarily involves the left side of the heart and is associated with mortality rates ranging from 25% to 40% [27]. Cure rates for right-sided *S. aureus *endocarditis in IDUs are high (>85%) and may be achieved with relatively short courses of treatment [27]. A case of community-acquired *S. aureus * tricuspid valve endocarditis with metastatic abscesses in the lungs and subcutaneous tissues in a patient with no obvious predisposing conditions is presented here.

## 2. Case Presentation

A 49-year-old male, with no previous medical history presented to our emergency room (ER) with generalized body pain that started 7 days prior to the visit. This was followed, two days later, by a high-grade fever associated with chills and rigors. *During the week prior to his presentation to the ER*, he noticed some swellings over the right upper chest and left calf that made it difficult for him to walk. The pain and fever improved with nonsteroidal anti-inflammatory drugs (NSAIDs) while the swelling continued. Later his urine became dark colored and he also noticed that his eyes *had* turned yellow. Review of other systems was negative for any major complaint. He was not on any long-term medications and has denied any illicit intravenous drug use (IDU) or any surgical or dental procedures.

Physical examination at the ER revealed a middle-aged man who did not appear in any form of distress, febrile (39°C), not pale, but icteric. His other vital signs were normal. Warm, tender, and fluctuant swellings were noticed over the right wrist, both calves, and below the right clavicle. All other aspects of the physical examination were normal. There were no peripheral stigmata of IE, venipuncture marks, heart murmurs, or lymphadenopathy.

**Figure 1:**
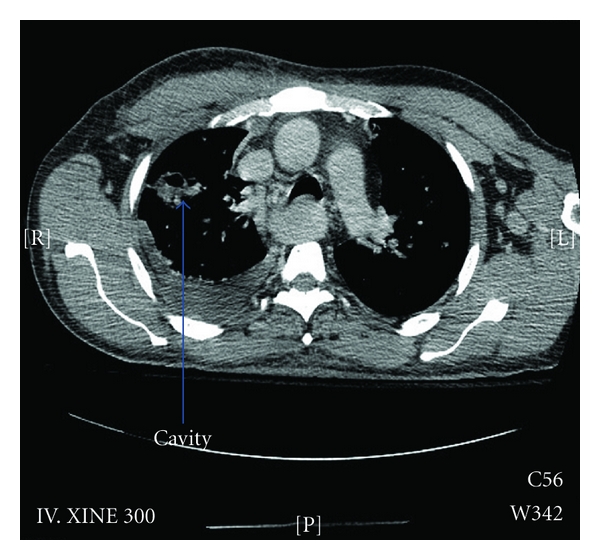
CT scan of the chest showing multiple abscess cavities.

**Figure 2:**
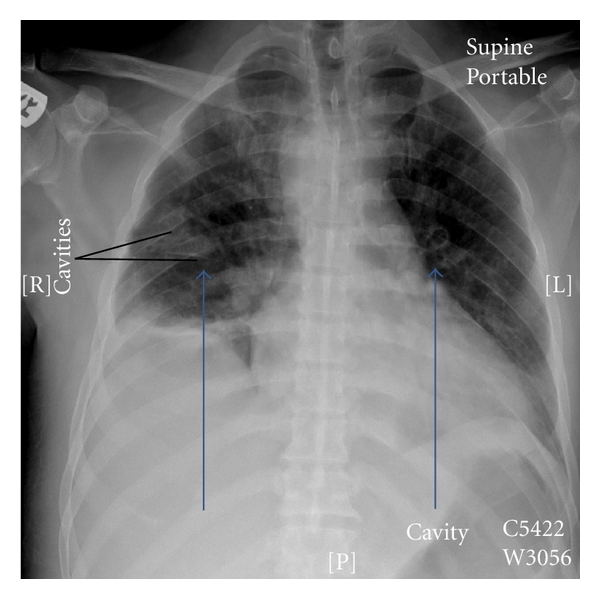
Chest X-ray of the chest showing multiple abscess cavities.

*Investigations including aspirates from the abscesses for culture and sensitivity were taken for culture and sensitivity*. Initial laboratory results revealed the following: total WBC-18.5 × *10* ^*9*^ */*L (with a left shift), platelet count-48,400, hemoglobin level-13.4 g/dL, BUN-25.1, serum creatinine-237 *μ*mol/L, ALT-111, AST-118, alkaline phosphatase-271, gamma GT-133, LDH-316, albumin-17, and total bilirubin-129, (mainly direct-109), with normal serum electrolytes. Imaging of the limbs, chest, abdomen, and pelvis revealed multiple abscess collections in the calves, lungs, (Figures 1 and 2), and the right gluteal region. He also had right basal atelectasis and right minimal pleural effusion. He was empirically started on broad spectrum coverage for the preliminary Gram-positive cocci (GPC) in clusters that were growing from the peripheral blood and aspirates from the abscesses. The empiric antibiotics included vancomycin and cloxacillin, both given intravenously (IV). The vancomycin was later discontinued (and cloxacillin continued) when the final culture and sensitivity results from the peripheral blood and multiple abscesses yielded heavy growth of methicillin-sensitive *Staphylococcus aureus* (MSSA). Gentamicin was added to the cloxacillin for the first fourteen days. He was also started on anticoagulant therapy for the suspicion of PE; however, this was stopped after the spiral CT was returned negative for PE. 

Other laboratory tests revealed: C-reactive protein-297 mg/dL, erythrocyte sedimentation rate (ESR) of 75 mm in the first hour, and D-dimer level of 1100. Serologic tests for HIV1 and HIV 2, HBsAg, and anti-HCV were all negative. Spiral computed tomography (CT) scan of the chest was not suggestive of pulmonary embolism (PE) and endoscopic retrograde cholangiopancreatography (ERCP) was also negative. Transthoracic echocardiography (TTE) and transesophageal echocardiography (TEE) revealed a large mass attached to both septal and anterior tricuspid valve leaflets, the biggest being 21 mm in diameter; however, there was no tricuspid regurgitation.

The patient's management was later transferred under the infectious diseases (ID) service from the gastroenterology unit under whom he was earlier admitted due to the elevated liver enzymes and jaundice. During his hospital stay, he had recurrent bouts of high fevers with chills and rigors, tachycardia, shortness of breath, diaphoresis, and feeling of impending doom with panic attacks and hypoxemia that necessitated repeated chest imaging with no evidence of PE. The abscesses were incised and drained, and the wounds managed by wound care specialists with complete healing. Due to the initial presentation, the patient was referred to various specialties, including psychiatrists at different times during the course of his hospital stay.

As his condition improved, the fever subsided, the jaundice cleared and the abscesses healed. Repeat blood culture became negative within 24 hours. Complete blood count (CBC) liver enzymes and bilirubin levels renal function and coagulation profile all normalized before his discharge.

The patient made remarkable improvement without any new or recurrent problems and was discharged home after 6 weeks of intravenous cloxacillin therapy and given appointment to be seen in the ID clinic after 4 weeks.

## 3. Discussion

Isolated native TVE secondary to SAB without any documented risk factors has continued to develop, eluding diagnosis and often discovered late or only at autopsy [9, 10, 21]. Infection of the right heart valves appears in 5%–10% of all cases and is almost always associated with intravenous drug abuse, with more than 80% of tricuspid valve endocarditis cases occurring in drug addicts [27]. Community acquisition was associated with a rapidly progressive course and often complicated with respiratory insufficiency, mimicking heart failure or pulmonary embolism [28]. Tricuspid valve involvement in a patient with no predisposing conditions and a structurally normal heart, as occurred in our patient, is a clinical rarity in which the diagnosis can be quite challenging.

Recurrent chest symptoms (e.g., shortness of breath, feeling of choking with hypoxemia), febrile episodes, and feeling of impending doom following wound dressing have recurred repeatedly in our patient. The constellation of these chest symptoms in this group of patients is termed as “the tricuspid syndrome” by some authors, and became less prominent as the wounds healed. This was initially thought to be due to pulmonary embolism or a form of psychiatric presentation. However, spiral CT of the chest and empiric therapy for pulmonary embolism and antipsychotic medications were to no avail. Association of these features with wound manipulation was later considered to be due to what some authors called “the tricuspid syndrome,” and it got less prominent as the wounds healed. This entity usually has a good prognosis with good response to medical therapy, and presents certain common clinical features (persistent fever, pulmonary lesions, anemia, and microscopic hematuria) that can lead the clinician to suspect the diagnosis. Some of these manifestations are thought to be due to an immunological mechanism [29].

Our patient presented with metastatic abscesses in the lungs that led to repeated episodes of shortness of breath with hypoxemia and was initially thought to have pulmonary embolism. This form of presentation was found to be significantly more common with community-acquired compared with hospital-acquired SAB (43 versus 21 percent) [1]. Community-acquired SAB complicated by TV IE usually has a better outcome than hospital-acquired infection though Nolan and Beaty [15] reported that up to 60% (63/105) of their patients had a mortality rate of 21% [30]. In a recent study by Hill et al, 6% of their patients with SAB had IE and they concluded that independent predictors of IE among patients with SAB included unknown origin of bacteremia, presence of a valvular prosthesis, persistent fever, and persistent bacteremia. The strongest predictor of complicated SAB was found to be a positive follow-up blood culture result at 48–72 hours [13]. Four risk factors (community acquisition, skin examination findings suggesting acute systemic infection, persistent fever at 72 hours, and positive follow-up blood culture results at 48 to 96 hours) have been found to accurately identify complicated SAB. Our patient had three of these risk factors.

**Figure 3:**
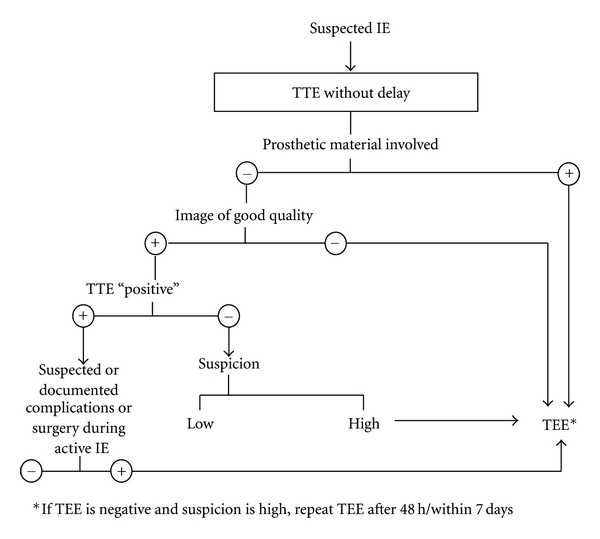
Algorithm for the use of transthoracic (TTE) and transesophageal echocardiography (TEE) in suspected IE. Note: TTE “positive” indicates finding typical of IE (e.g., fresh vegetation or abscess formation).

With the recurrent chest symptoms and a positive blood culture for *Staphylococcus aureus* bacteremia, a TEE was performed according to current guidelines [31, 32], and multiple vegetations were identified (Figure 3). All patients with SAB should undergo a TEE irrespective of whether a risk factor for IE has been identified or not to avoid the uncertainties that plagued previous studies [30].

The index case was treated with intravenous cloxacillin, and also additionally received gentamicin for the first two weeks for its synergistic effect; then cloxacillin was continued for the remaining 4 weeks. The benefit of gentamicin in native valve endocarditis lies in earlier defervescence of fever and the sterilization of blood cultures; there is no proven survival advantage. Our patient remained on admission for six weeks for intravenous antibiotic therapy with favorable outcome. Before discharge, his temperature, blood counts, coagulation profile, renal, and hepatic functions *had* normalized without additional interventions. Several studies have reported the benefits of combined *β*-lactam/aminoglycoside short-course (2 weeks) therapy in patients with complicated *Staphylococcus aureus* endocarditis (with evidence of renal failure and extrapulmonary metastatic foci) [33–37]. *This study confirms this*. By contrast, glycopeptide (teicoplanin or vancomycin) plus gentamicin-based short-course regimens appeared to be less effective for right-sided *S. aureus *IE caused by oxacillin-susceptible *S. aureus *(OSSA) [30]. Glycopeptides are intrinsically less active against staphylococci than are antistaphylococcal *β*-lactams [28, 29].

Prognosis of tricuspid valve endocarditis is favorable, and most cases respond to antibiotic therapy [38]. However, surgical treatment should be considered for those with severe congestive heart failure, persistent sepsis, development of abscesses, and mycotic endocarditis. Other major indications for surgery will include the presence of large tricuspid valve vegetations (>1 cm) with persistent fever, tricuspid valve insufficiency or pulmonary embolization [39, 40]. Our patient did well on medical therapy alone, although surgery was considered an option at the outset.

We suggest that right-sided endocarditis must be considered in any patient with the “tricuspid syndrome,” with recurrent pulmonary events, anemia, and microscopic hematuria. Careful evaluation of prior medical records and clinical course can be very helpful. Echocardiography and serial blood cultures provide the key to diagnosis.
